# A nomogram for preoperative prediction of lymphatic infiltration in colorectal cancer: A personalized approach to clinical decision making

**DOI:** 10.1097/MD.0000000000018498

**Published:** 2019-12-27

**Authors:** Guo Wu, Jun-Gang Liu, Xiao-Liang Huang, Chun-Yin Wei, Franco Jeen PC, Wei-Shun Xie, Shao-Mei Chen, Chu-Qiao Zhang, Wei-Zhong Tang

**Affiliations:** a Department of Gastrointestinal Surgery, Guangxi Medical University Cancer Hospital; b Guangxi Clinical Research Center for Colorectal Cancer, Nanning, Guangxi Zhuang Autonomous Region, P.R. China.

**Keywords:** colorectal cancer, lymphatic infiltration, nomogram, prediction model, risk stratification

## Abstract

Lymphatic infiltration (LI) is a key factor affecting the treatment of patients with colorectal cancer (CRC). Thus, the aim of this study was to develop and validate a nomogram for individual preoperative prediction of LI in patients with CRC.

We conducted a retrospective analysis of 664 patients who received their initial diagnosis of CRC at our center. Those patients were allocated to a training dataset (n = 468) and a validation dataset (n = 196). The least absolute shrinkage and selection operator regression model was used for data dimension reduction and feature selection. The nomogram was constructed from the training dataset and internally verified using the concordance index (C-index), calibration, area under the receiver operating characteristic curve and decision curve analysis (DCA).

The enhancement computed tomography reported N1/N2 classification, preoperative tumor differentiation, elevated carcinoembryonic antigen, and carbohydrate antigen19-9 level were selected as variables for the prediction nomogram. Encouragingly, the nomogram showed favorable calibration with C-index 0.757 in the training cohort and 0.725 in validation cohort. The DCA signified that the nomogram was clinically useful. The Kaplan–Meier survival curve showed that patients with LI had a worse prognosis and could benefit from postoperative adjuvant chemotherapy.

Use common clinicopathologic factors, a non-invasive scale for individualized preoperative forecasting of LI was established conveniently. LI prediction has great significance for risk stratification of prognosis and treatment of resectable CRC.

## 1 Introduction

Not only is colorectal cancer (CRC) the third most common malignancy but it also ranks as the fourth leading cause of cancer-related deaths worldwide.^[1]^ Surgery remains the mainstay of curative treatment and the attention has been primarily focused on prognosis and outcome of patients with CRC.[^2^,^3^,^4^,^5^,^6^,^7^] Lymphatic infiltration (LI) is an important parameter of the routine pathological report after resection of CRCs. The 8th edition of the guidelines for CRC recommended by the National Comprehensive Cancer Network (NCCN), states that evidence-based medical evidence suggests that nonmetastatic rectal cancer or colon cancer above T3 with high-risk factors for lymph nodes can benefit from preoperative neoadjuvant therapy.^[8]^ The European Society for Medical Oncology (ESMO) recommends that, for nonmetastatic colon cancers stage ≥ T2N0M0, the related lymphatic drainage requires removal while a wide resection of the involved segment of bowel is performed.^[9]^ Predictive values of LI can be used to identify tumors with occult lymph node metastasis,^[10]^ which are firmly related to prognosis and aid in clinical decision-making usefully. Thus, an accurate identification of preoperative LI in CRC is crucial to prognosis and treatment strategy decisions.

A nomogram is a graphical calculation scale, while least absolute shrinkage and selection operator (LASSO) is a regression analysis method. The combination of the 2 can contribute to quantifying the individual risk of a particular outcome in a variety of cancers reliably and pragmatically.^[11]^ However, nomograms for prediction of preoperative LI in CRC patients have been relatively few to date, but are promising. The LI is also a promising predictive factor available in the nomogram. Particularly, it is necessary to develop predictive nomograms that can serve as a useful guide in patient management. In the future, the accumulation of these data could serve as evidence to identify patients who should receive additional chemotherapy or radiation therapy versus those who can avoid over-treatment.

## 2 Material and methods

### 2.1 Patients

The study protocol was approved by the Ethics Committee of Affiliated Tumor Hospital of Guangxi Medical University (No. LW2018037). All relevant data and materials are available. Permission to obtain the data can be requested by E-mail. We enrolled 664 CRC patients who underwent curative surgery in the department of gastrointestinal surgery at the Cancer Hospital of Guangxi Medical University between August 2013 and April 2018. Inclusion criteria included the following:

(1) pathologically confirmed CRC patients.
(2) primary tumor resection.
(3) availability of postoperative pathology reports for LI.[^12^,^13^]

Exclusion criteria included the following:

(1) any preoperative treatment (including radiotherapy, chemotherapy, or chemoradiotherapy),
(2) patients with other neoplastic disease during the same period, and
(3) familial adenomatous polyposis or hereditary colon cancer.[^12^,^13^]

Baseline clinicopathologic parameters, including age, gender, body mass index, past and family history, preoperative and postoperative blood routine examination, serological markers, enhanced computed tomography (CT)-based TNM classification, the degree of preoperative histological differentiation and gross type of tumor were derived from the medical records. The evaluation of the tumor pathologic staging was performed on the basis of the Union for International Cancer Control 8th edition TNM staging system.^[12]^ All 664 patients were randomized into 2 independent datasets according to computer-generated random numbers in a proportion of 7:3 (468 cases in the training dataset and 196 cases in the validation dataset).

### 2.2 Feature selection and development of an individualized prediction model

The LASSO method was used for data dimension reduction and promising feature selection based on training dataset.[^14^,^15^] All categorical variables were converted to dummy variables. The dependent variable was the state of the LI. The suitable tuning parameter (*λ*) for the LASSO logistic regression was determined using cross-validation. The goodness of fit between observed event rates and predicted values was assessed by calibration curve and examined by Hosmer–Lemeshow test. The Pearson Chi-squared goodness of fit test confirmed that the observed proportions matched expected proportions significantly. The individualized prediction model was testified by using the receiver operating characteristic (ROC) curve and the area under the curve (AUC). The ROC, known as a relative operating characteristic curve, was used to compare the true positive rate (TPR) and the false positive rate as the criterion changes.^[16]^ In the logistic regression model, the AUC is equal to the C-index. Both value of AUC and ROC vary between 0 and 1, where 0 represents chance performance, while 1 represents perfect performance.^[17]^

### 2.3 Validation of the nomogram and clinical utility

Internal validation was performed using validation dataset which was randomly extracted from the population and accounted for 30% of the total patient population. The individualized prediction model was evaluated via the calibration curve and Hosmer–Lemeshow test in the validation cohort. Decision curve analysis (DCA) determined the clinical usefulness of the nomogram by quantifying the net benefits at different threshold probabilities in the combined training and validation dataset.

### 2.4 Statistical analysis

Patients were randomly assigned to either the training cohort or the validation cohort. Descriptive statistics were incorporated from the medical records. All statistical analyses were performed using R software (version 3.4.0). The LASSO logistic regression analysis was performed using the “glmnet” package. Use the “rms” package for logistic regression analysis, nomogram plots, and nomogram calibration. The DCA was performed using the “dca.R” function and the Hosmer–Lemeshow test was performed using the “HLtest.R” function. The differences were statistically significant at the 2-sided *P* value <.05.

## 3 Results

### 3.1 Clinical characteristics

We enrolled 664 CRC patients who did not undergo adjuvant therapy before surgery from August 2013 to April 2018. The clinical parameters of the development and validation cohorts are presented in Table 1. Patients had a mean age of 59.2 years (range 17–87 years). The approximate rate of male to female was 1.414:1 and about a half were rectal cancer. Furthermore, over 80% of the patients under the colonoscopy found that the degree of tumor differentiation is moderate. The carcinoembryonic antigen (CEA) and carbohydrate antigen (CA19-9) levels were measured at the time of admission. The threshold value for CEA level was 5 ng/mL and for CA199 was 37 U/mL, which were consistent with other promulgated articles.[^4^,^18^]

**Table 1:**
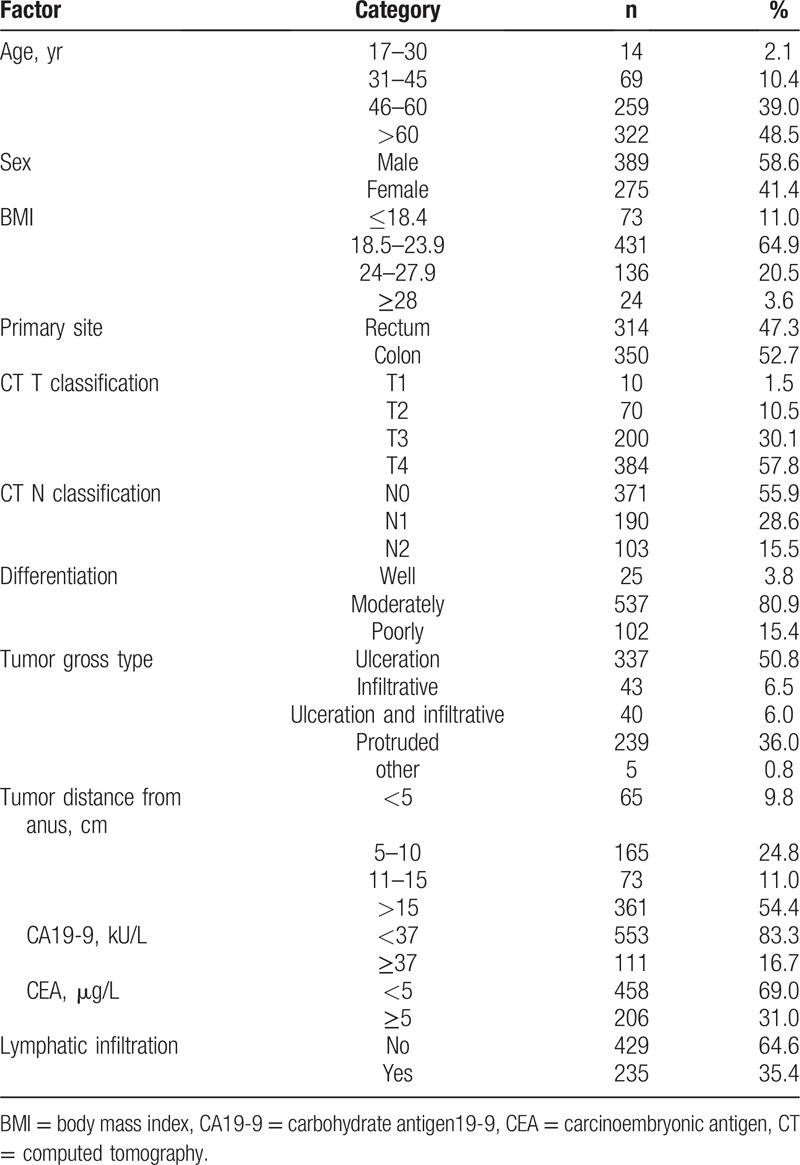
Characteristics of patients with colorectal cancer.

### 3.2 Feature selection

**Figure 1:**
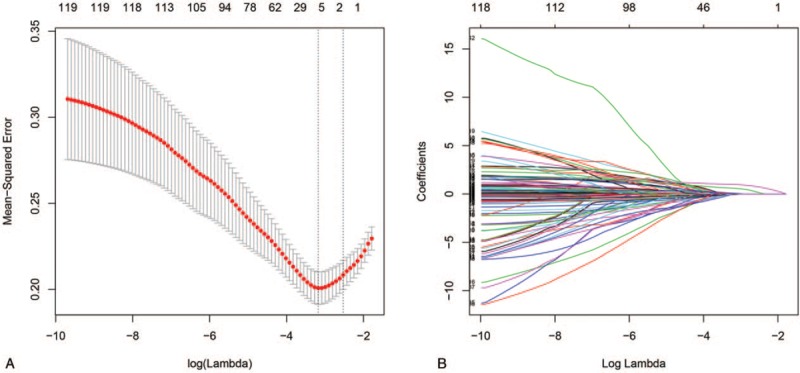
Feature selection using LASSO logistic regression. (A) Tuning parameter (*λ*) selection in the LASSO logistic regression used 10-fold cross-validation via minimum criteria. The binomial deviance was plotted versus log (*λ*). The black vertical lines were plotted at the optimal *λ* based on the minimum criteria and 1 standard error of the minimum criteria. (B) LASSO coefficient profiles of the 119 clinical features. A coefficient profile plot was produced versus the log (*λ*). LASSO = least absolute shrinkage and selection operator.

**Figure 2:**
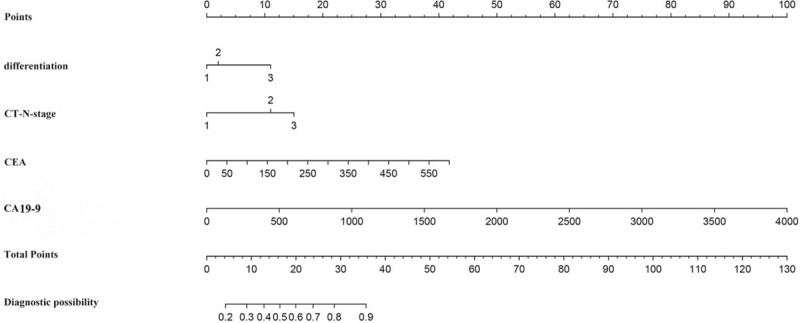
Nomogram for preoperative prediction of lymphatic infiltration in CRC. The nomogram was developed in the primary cohort, with the differentiation, CT reported N classification, CEA and CA19-9 incorporated. CA19-9 = carbohydrate antigen19-9, CEA = carcinoembryonic antigen, CRC = colorectal cancer, CT = computed tomography.

The most significant predictive markers were selected via the training dataset by LASSO logistic regression algorithm and contributed powerfully to the final prediction model. A total of 119 features were used for the LASSO logistic regression, and 4 features with non-zero coefficients were subsequently selected, with an optimal lambda value of 0.042 (Fig. 1A and B). The model ultimately included 4 features: the enhancement CT-based N status, preoperative histological grade, and the elevated CEA and CA19-9 levels (Fig. 2).

### 3.3 Nomogram construction and performance assessment

The 4 features selected using the LASSO logistic regression algorithm were engaged in the multivariate logistic regression modeling. With 4 independent prediction points assigned in each horizontal segmentation, a vertical line is drawn from the 4 rows above to sum the total scores. The corresponding relationship between the total score and the probability of LI was used to calculate the risk of each patient. Multivariate logistic regression revealed that LI was independently influenced by enhancement CT-based N1 status (*P* = 1.11 × 10^-7), CT-based N2 status (*P* = 6.14 × 10^-8), CA19-9 level (*P* = .021), poor differentiation (*P* = .058), and CEA level (*P* = .090) in Table 2.

**Table 2:**
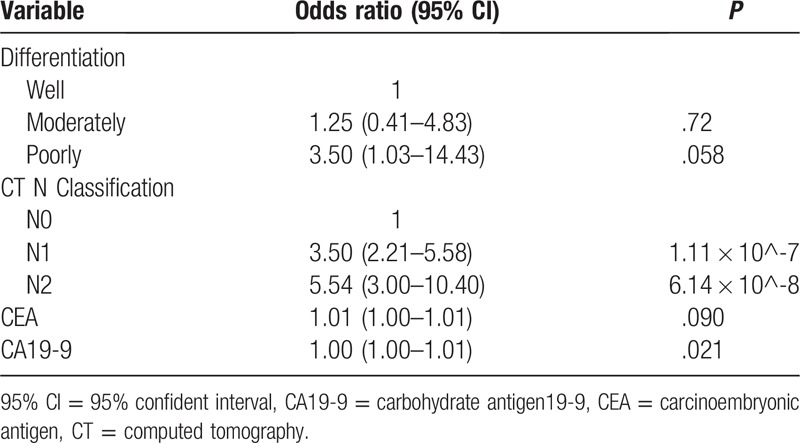
Risk factors for lymphatic infiltration in colorectal cancer.

**Figure 3:**
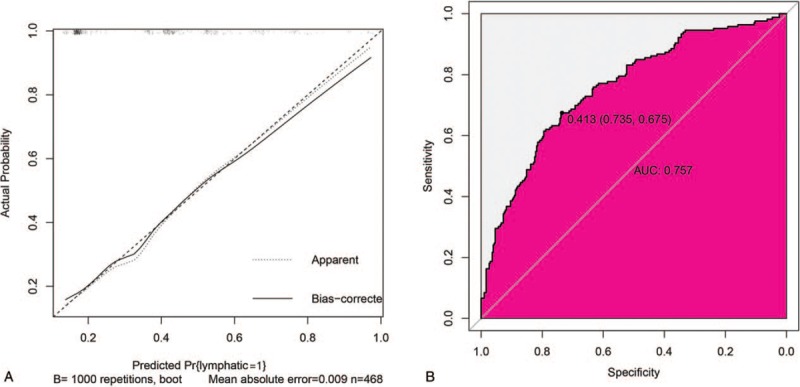
The performance of nomogram in training dataset. (A) The calibration plot of the nomogram in the training dataset. The *x*-axis is nomogram-predicted probability of lymphatic infiltration and *y*-axis is actual lymphatic infiltration. The reference line is 45° and indicates perfect calibration. (B) The ROC curves of the nomogram in the training. ROC = receiver operating characteristic.

The calibration plot demonstrated favorable agreement between the predicted and observed values in the training dataset (Fig. 3A). Hosmer–Lemeshow test identified the data as non-significant (*P* = .45), indicating that the deviation is not fully fit. The C-index for the prediction nomogram in the primary cohort was 0.757 (Fig. 3B).

### 3.4 Validation of the nomogram

**Figure 4:**
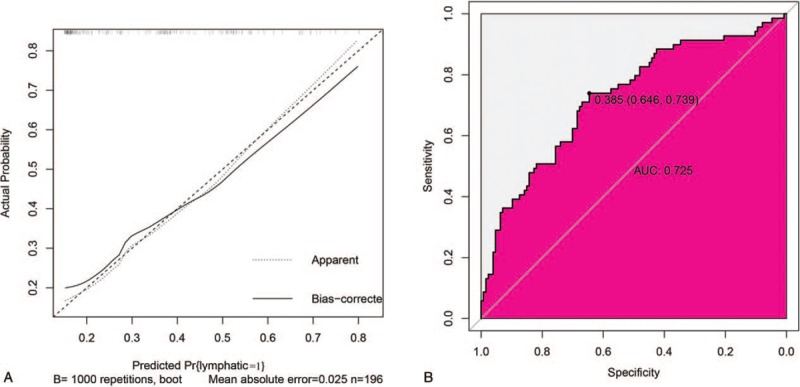
The performance of nomogram in validation dataset. (A) The calibration plot of the nomogram in the validation dataset. The x-axis is nomogram-predicted probability of lymphatic infiltration and *y*-axis is actual lymphatic infiltration. The reference line is 45° and indicates perfect calibration. (B) The ROC curves of the nomogram in the validation dataset. ROC = receiver operating characteristic.

The internal validation was used to test and verify the nomogram using 196 patients randomly selected from the original pool of 664 patients. The predicted and observed outcomes were again very similar and were shown in Figure 4A. The Hosmer–Lemeshow test displayed no lack of fit with a *P*-value of .14 (Fig. 4A) and the AUC of the validation nomogram turned was 0.725 (Fig. 4B).

### 3.5 Clinical utility of the nomogram

**Figure 5:**
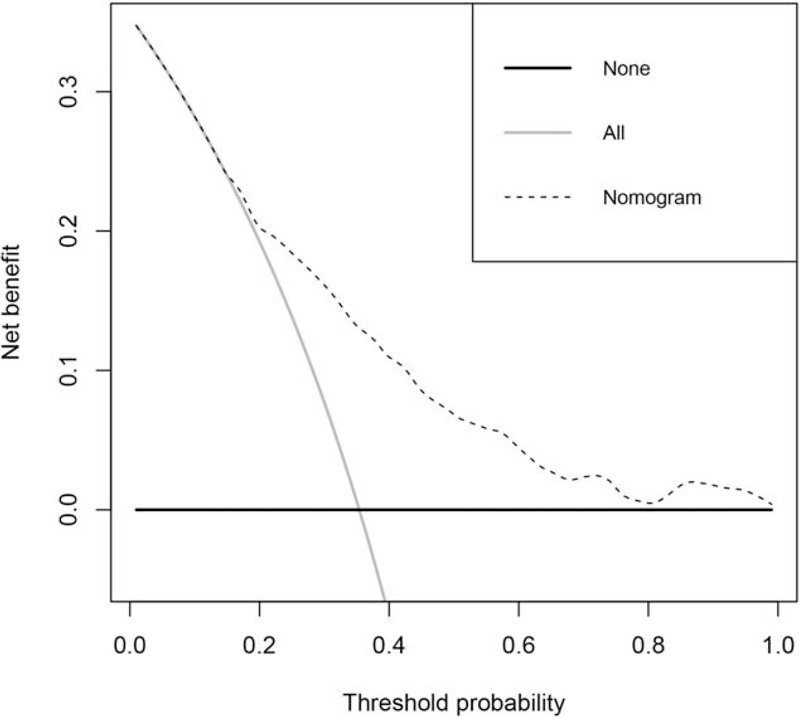
DCA curve for the nomogram. The net benefit was plotted versus the threshold probability. The dotted line represents the nomogram. The gray and black lines represent the treat-all-patients scheme or the treat-none scheme, respectively. DCA = decision curve analysis.

The net benefit was plotted versus the threshold probability (Fig. 5). The decision curve showed that if the threshold is >16% and <80%, the nomogram to predict LI is more beneficial than using a total treatment patient regimen or no treatment regimen. For example, the nomogram added a net benefit of 12.5% at a certain probability of 35%, which indicates the significant clinical use of this nomogram.

### 3.6 Prognostic analysis

**Figure 6:**
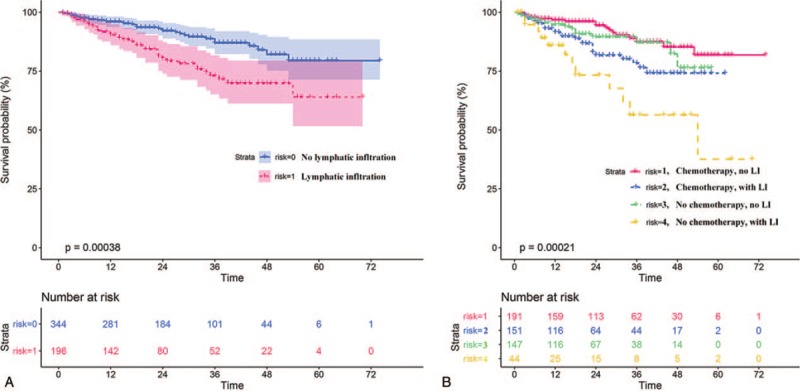
The Kaplan–Meier survival curve. (A) The prognosis of CRC patients with or without lymphatic infiltration. (B) The subgroup analysis took those patients into 4 groups based on postoperative adjuvant chemotherapy. CRC = colorectal cancer.

The Kaplan–Meier survival curve showed that patients with LI had a worse prognosis (*P* = 3.8 × 10^−4^) which meant LI had a meaningful relationship with poor prognosis for CRC patients (Fig. 6A). What’ more, when took postop chemotherapy into consideration, we observed that, among patients with lymphatic invasion, patients who received adjuvant chemoradiotherapy had a better prognosis than patients who did not receive adjuvant chemoradiotherapy (*P* = .0425). However, among patients without LI, there was no significant difference in overall survival (OS) between patients with or without adjuvant chemoradiotherapy (*P* = .3645, Fig. 6B). In other words, patients with no LI had no obvious survival benefit after postoperative adjuvant chemotherapy.

## 4 Discussion

We report here the development and validation of a nomogram to predict preoperative LI based on the preoperative clinical features of CRC patients at our center. The nomogram provided favorable discrimination and calibration values. To our knowledge, this is one of the only few studies that predict LI in such a large CRC patient population. As for CRC above T3 with a high-risk of LI receiving neoadjuvant therapy before surgery to achieve tumor degeneration and reduce the risk of postoperative recurrence. In rectal cancer, after T1N0 tumor resection through the anus, if there are high-risk factors present such as T2, LI, further rescue radical surgery or radiotherapy or chemotherapy are recommended, the preoperative LI nomogram can distinguish the high-risk group to avoid two traumas. It was reported by the Tumor Center Regensburg that the risk of T1 rectal cancer with regional lymph node metastasis is about 6.9%.^[19]^ Endoscopic resection cannot confirm the extent of lymph node involvement and there is no sensitive or specific means to assess the risk of lymph node metastasis. Regional lymphatic metastasis affects tumor stage and the corresponding treatment. Fernando et al^[20]^ established a predictive model for T1 CRC that lymph node metastasis was indeed associated with LI, which makes the model a possible supplement for those understaged patients.[^21^,^22^]

Lymphatic vessel infiltration have been considered as a poor prognostic marker for recurrence and survival in stage II and stage III patients.[^23^,^24^] The Kaplan–Meier survival curve emphasizes that patients with LI have a much worse prognosis if no postoperative chemotherapy is carried out (Fig. 6). LI has been recognized as a significant risk factor for lymph node metastasis of CRC,^[25]^ which is an important mechanism of spreading and its presence is associated with poor prognosis for OS and disease-free survival.^[26]^ LI was demonstrated in 35.4% of our 664 patients, which is more than the 14.1% displayed by Durante et al^[27]^ and 15% from the Swedish colorectal cancer registry.^[28]^ It may be related to the accuracy of the equipment or the increased value of screening, but mostly blame on the current medical resources and the environment in China.

It is not surprising that CT-reported N1/N2 classification, poor differentiation, and elevated CEA and CA19-9 levels increased the risk of LI, which were basic route in the diagnostic criteria and treatment of CRC guided by the NCCN.^[29]^ Furthermore, the nomogram consisted of readily available factors that are subject to little interobserver variability. CT is a relatively accurate and reliable detection and diagnosis method for CRC with a sensitivity of 78.6% and specificity of 75%.[^30^,^31^] In the present study, the CT-reported N classification (*P* = 1.11 × 10^-7) was identified as a major predictive factor, which is in line with several influential cohorts; for example, Huang at al^[18]^ built a radiomics nomogram which incorporated CT-reported N status (*P* < .001) as an independent clinicopathologic risk factor. CEA and CA19-9 levels have been extensively used for clinical diagnoses of CRC with both sensitivities up to 72% and high specificity.^[32]^ Furthermore, poorly differentiated pathology and CEA >5 ng/dL has been considered high-risk for recurrence in classification II colon cancer by the American Society of Clinical Oncology and ESMO.^[33]^ Previous reports have found that patients with high scores of same above prognostications could benefit from adjuvant chemotherapy.[^34^,^35^] In addition, existing studies have shown that CEA level could serve as an important marker for prognosis and risk-benefit discussion for adjuvant chemotherapy.^[34]^ In this era of precision medicine, it is very convenient for clinicians to acquire the easy applicability of a forecasted score that identifies long-term prognosis.

In the scale of the nomogram, even if N2 classification got higher points than N1 classification, but when opposed to other factors shown in the figure seemed little difference between the two classifications. What's more, poorly differentiated tumors had a higher risk of LI than moderate- or well-differentiated ones. More than 80% of our patient's diagnoses were moderate degree of differentiation, and, according to enhanced CT, approximately 55% patients did not have lymph node metastasis. Ultimately, the score of lymph node classification and tumor differentiation provided a baseline, while tumor markers identified a high-risk for LI.

The key strength of our study is the use of data from a large sample of patients using only common clinicopathological factors, which permit high generalizability. Despite recent reports of a radiomics group improving the accuracy in predicting OS,^[18]^ the practicality and universality are important problems. In addition, the prediction results are not much different. However, the weakness of the present study is that external validation could be more convincing as the patients were from a single center. Despite this, we still believe that our nomogram could detect LI in CRC accurately and consistently. It is straightforward to implement, easy to understand, and the results can be used to help inform the risk-benefit discussion.

## 5 Conclusions

We use common clinicopathological factors to build a preoperative LI prediction nomogram to help the treatment and risk stratification of prognosis of patients with CRC.

## Acknowledgments

We wish to that the doctors in the imaging and pathology departments of our hospital for their help. We would like to thank Editage (www.editage.com) for English language editing. Thanks to each member of our team in this series of studies.[^36^,^37^]

## Author contributions

**Conceptualization:** Guo Wu, Jun-Gang Liu, Xiao-Liang Huang, Shao-Mei Chen, Chu-Qiao Zhang, Wei-Zhong Tang.

**Data curation:** Guo Wu, Franco Jeen P C, Wei-Shun Xie, Shao-Mei Chen, Chu-Qiao Zhang.

**Formal analysis:** Jun-Gang Liu, Xiao-Liang Huang, Chun-Yin Wei, Shao-Mei Chen, Chu-Qiao Zhang.

**Investigation:** Guo Wu, Jun-Gang Liu, Chun-Yin Wei, Wei-Zhong Tang.

**Methodology:** Guo Wu, Jun-Gang Liu, Xiao-Liang Huang, Chun-Yin Wei, Wei-Shun Xie, Wei-Zhong Tang.

**Project administration:** Guo Wu, Jun-Gang Liu, Xiao-Liang Huang, Chun-Yin Wei, Wei-Shun Xie, Wei-Zhong Tang.

**Software:** Xiao-Liang Huang.

**Validation:** Guo Wu, Jun-Gang Liu.

**Visualization:** Franco Jeen P C.

**Writing – original draft:** Guo Wu.

**Writing – review and editing:** Guo Wu, Franco Jeen P C, Wei-Zhong Tang.
